# Association of Sleep Duration With Serum Estradiol Concentrations Among American Men and Women: Evidence From NHANES 2013–2016

**DOI:** 10.1155/ije/7863420

**Published:** 2025-02-07

**Authors:** Zhisheng Zhu, Shiquan Wu, Xingong Lin, Chaoyang Wang, Xianying Zhou

**Affiliations:** Plastic Surgery, The Second Affiliated Hospital of Fujian Medical University, Quanzhou, China

**Keywords:** age, NHANES, serum estradiol, sleep

## Abstract

**Objective:** To evaluate the association between sleep duration with serum estradiol concentrations and its variation by sex and age in American adults.

**Methods:** Data were analyzed for 5406 men and women (≥ 20 years old) who participated in the cycles of the National Health and Nutrition Examination Survey 2013–2016, a cross-sectional study. Total estradiol (pg/mL) was measured and categorized (low, normal, and high) based on the NHANES protocol. Sleep duration was classified as ≤ 6, 6–9, and ≥ 9 h. Weighted multivariable adjusted and multinomial logistic regression models were conducted to assess these associations.

**Results:** Our multivariable multinomial logistic regression analysis revealed no significant associations between sleep duration and serum estradiol concentrations among both American men and women. Specifically, comparisons of sleep durations (≤ 6 and ≥ 9 h) to the reference group (6–9 h) across various age categories showed odds ratios for low and high estradiol concentrations that remained statistically nonsignificant in fully adjusted models. These findings suggest that, unlike previous studies linking sleep duration with variations in other hormones, estradiol concentrations do not appear to be significantly affected by differences in sleep duration in either sex across all age groups studied.

**Conclusion:** The lack of significant associations between sleep duration and serum estradiol concentrations indicates that sleep duration may not influence estradiol levels in the general population of American men and women. These results underscore the importance of continued research into how sleep influences hormonal balance. However, it is important to note that the NHANES data we used are from a cross-sectional study, which cannot establish a causal relationship between sleep duration and serum estradiol. Future studies should investigate additional factors, such as genetic predispositions, lifestyle habits, and environmental influences, that may modulate the relationship between sleep and hormone levels.

## 1. Introduction

Sleep is a fundamental biological process, occupying about one-third of human life. The American Academy of Sleep Medicine, the Sleep Research Society, and the National Sleep Foundation recommend that adults get at least 7 h of sleep per night regularly [1]. The global prevalence of insomnia is estimated to be around 10%–30%, with rates as high as 50% among midlife women [2]. Scientific reviews suggest that sleep disorders or alterations in sleep duration and timing may impact reproductive health, including fertility, though the biological mechanisms remain unclear [3].

Estradiol is a neuroactive steroid, with estrogen receptors present in sleep and arousal-regulating nuclei, such as the preoptic area of the hypothalamus, the suprachiasmatic nucleus, and the locus coeruleus [4]. Therefore, estradiol, along with other hormones of the hypothalamic–pituitary–ovarian axis, may influence sleep–wake regulation directly. For example, it might affect sleep homeostasis by influencing adenosinergic actions in the preoptic area (a sleep-promoting nucleus) or by modulating arousal systems in the lateral hypothalamus (rich in hypocretin-releasing neurons that promote wakefulness) or the locus coeruleus (a primary site of norepinephrine involved in arousal) [4, 5].

Current research on the association between sleep duration and serum estradiol concentrations mainly focuses on adult women. This may be due to the higher risk of insomnia or insufficient sleep in women compared to men, which could be related to their unique gonadal hormone environment. Estradiol, the primary estrogen in women of reproductive age, plays a crucial role in ovulation, follicular growth and development, and the maintenance of female secondary sexual characteristics. Additionally, the dramatic changes in ovarian hormone levels during puberty, pregnancy, or menopause in adult women contribute to the controversy in current research findings on the association between sleep duration and serum estradiol concentrations. For example, the BioCycle study found that for every additional hour of daily sleep, the average estradiol concentration increased by 3.9% [6]. In contrast, a study on Japanese pregnant women found that maternal estradiol levels were negatively correlated with weekend sleep duration [7]. Research on this association in men has also yielded inconsistent results. In adult men, some studies reported no association between sleep duration and estradiol levels [8]. However, in adolescent males, higher estradiol levels were associated with poorer sleep quality [9]. These discrepancies in research findings may be related to age, sex, and menopausal status, but these differences remain inconclusive.

Therefore, considering the impact of age and sex on sleep duration and estradiol levels, we investigated whether the relationship between sleep duration and serum estradiol levels varies by age and sex. These findings could be significant for managing healthy sleep to maximize the quality of life and reproductive health for the American population.

## 2. Materials and Methods

### 2.1. Study Population

The National Health and Nutrition Examination Survey (NHANES) is a series of cross-sectional studies conducted in the United States aimed at assessing the health and nutritional status of adults and children. Distinguished from other studies, NHANES integrates physical examinations with interviews to amass comprehensive data. Each component of the study has been thoroughly reviewed and approved by the National Center for Health Statistics Ethics Review Board. Additionally, informed consent was obtained in writing from all participants.

**Figure 1:**
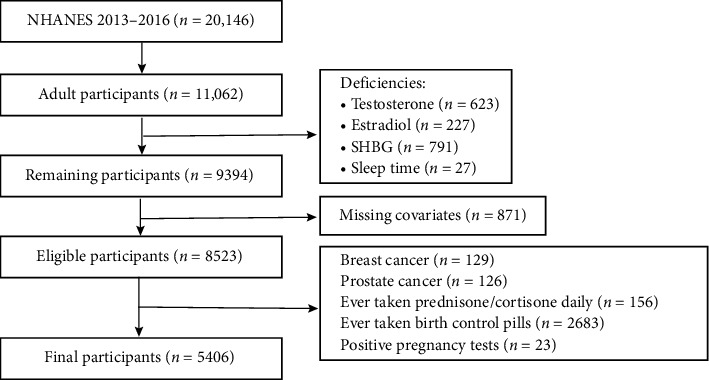
Flowchart for selecting the study population. NHANES, the National Health and Nutrition Examination Survey.

In this study, we utilized data from the NHANES for the years 2013–2016. Initially, 20,146 adults were identified, and those aged 20 years or older (*n* = 11,062) were selected for further analysis. In this study, we applied several exclusion criteria to ensure the accuracy and reliability of our findings. First, we excluded participants with missing data on estradiol, sleep duration, and important covariates (*n* = 2359). Additionally, we excluded individuals with a history of breast cancer (*n* = 129) and prostate cancer (*n* = 126), as certain treatments for these conditions may influence hormone concentrations [10]. Furthermore, participants who had taken glucocorticoids (such as prednisone and cortisone) daily (*n* = 156) were excluded due to the potential impact of these medications on the hypothalamic–pituitary–gonadal (HPG) axis, which could suppress gonadotropin secretion and affect estradiol synthesis. We also excluded individuals who had used oral contraceptives (*n* = 2683), as these medications typically contain synthetic estrogens and progestins that may alter endogenous estradiol levels through negative feedback mechanisms. Lastly, participants who tested positive for pregnancy (*n* = 23) were excluded, as their estradiol levels can significantly differ from those of nonpregnant individuals, potentially confounding the assessment of estradiol levels in our analysis. Ultimately, a total of 5406 participants were included in the analysis. A detailed depiction of the exclusion process is provided in Figure 1.

### 2.2. Assessment of Outcome—Testosterone, Estradiol, and Sex Hormone–Binding Globulin (SHBG)

In the NHANES study, the measurement of testosterone, estradiol, and sex hormone–binding globulin (SHBG) followed standardized laboratory procedures. For testosterone and estradiol, the key steps included dissociating the analytes from binding proteins, extracting them from the sample matrix, and quantifying them using isotope dilution high-performance liquid chromatography–tandem mass spectrometry (ID-LC-MS/MS). This method employed stable isotope-labeled internal standards and relied on liquid–liquid extraction for isolating the analytes, with measurements made using a triple quadrupole mass spectrometer [11].

For SHBG, the measurement technique involved an immunoassay with chemiluminescence detection. The process included two incubation steps, where the first involved using biotinylated- and ruthenium-labeled monoclonal antibodies against SHBG. In the second step, streptavidin-coated microparticles were added, which bound to the antibodies to form a complex. This complex was then measured chemiluminescently using a photomultiplier tube, with readings adjusted according to a specific calibration curve for each instrument and batch [11].

Following the protocols established by NHANES (Table S1), we defined normal reference ranges for total estradiol as follows: 10–50 pg/mL for adult males, 20–350 pg/mL for premenopausal adult females, and ≤ 20 pg/mL for postmenopausal females [12]. Additionally, conditions of “estradiol deficiency” and “excessive estradiol” were categorized based on deviations from these reference ranges.

### 2.3. Exposure Assessment: Sleep Duration

In the NHANES dataset, sleep duration was assessed using the question, “How much sleep do you usually get at night on weekdays or workdays?” where respondents could answer between 1 and 24 h. For the purpose of this analysis, sleep duration was divided into three categories in line with the guidelines issued by The American Academy of Sleep Medicine and Sleep Research Society: ≤ 6, 6–9, and ≥ 9 h [1]. The categories of ≤ 6 and ≥ 9 h were analyzed in comparison to the 6–9-h category (used as the reference group) to determine the odds of being in the estradiol deficiency or excessive estradiol categories.

### 2.4. Menopausal Status Definitions

In our study, the determination of menopausal status was based on two methods. First, we collected self-reported data from participants through a questionnaire. Specifically, participants were asked, “In the past 12 months, have you had at least one menstrual period?” (Please do not include bleeding due to medical conditions, hormone therapy, or surgery). For participants who answered “No,” NHANES staff further inquired, “What was the reason for not having a menstrual period in the past 12 months?” (Response options included “Pregnancy, breastfeeding, hysterectomy, menopause/life change, other, refused, don't know, missing”). We defined participants who answered “hysterectomy” or “menopause/life change” as being in a menopausal state [12]. All questions were administered by trained interviewers using a computer-assisted personal interview (CAPI) system at mobile examination centers to ensure data accuracy and consistency. Additionally, we also defined participants aged 55 years and older as being in a menopausal state. This criterion is based on the common definitions of menopause in the literature [13].

### 2.5. Assessment of Covariates

The selection of covariates in this study was informed by previous research on sex hormones [10, 14, 15] and included age (continuous), race (Mexican American, other Hispanic, non-Hispanic white, non-Hispanic black, Non-Hispanic Asian, or other race), testosterone level (continuous), SHBG level (continuous), education level (less than 9th grade, 9–11th grade, high school graduate/GED or equivalent, some college or AA degree, or college graduate or above), body mass index (BMI, continuous), diabetes (categorical), hypertension (categorical), session of blood sample collection (morning, afternoon, or evening), alcohol intake (1–5 drinks/month, 5–10 drinks/month, 10+ drinks/month, or nondrinker), smoking status (never smoker, former smoker, or current smoker), and physical activity (categorical).

To identify participants with diabetes, the following criteria were applied: (a) hemoglobin A1C concentration ≥ 6.5% or a fasting plasma glucose level ≥ 126 mg/dL; (b) affirmative answers to any of the questions: “Take diabetic pills to lower blood sugar?” “Doctor told you have diabetes?” or “Taking insulin now?” [16]. Participants visiting the mobile examination center provided three systolic and diastolic blood pressure readings. The mean of these readings was used for analysis, specifically identifying hypertension when the average was ≥ 140/90 mmHg. Additionally, the conditions “taking prescription for hypertension” or being “told by a doctor you have hypertension” were used as criteria for diagnosing high blood pressure [10]. Participants who currently smoke or have smoked more than 100 cigarettes in their lifetime are identified as current or former smokers, respectively.

### 2.6. Statistical Analysis

We used sampling weights to adjust for selection probabilities, oversampling, nonresponse, and demographic discrepancies between the sample and the entire U.S. population. We analyzed the distribution of sociodemographic and lifestyle factors across different sleep duration categories (Table 1). The representation of continuous variables was done as the mean ± standard deviation for normal distribution or as median (quartile) for skewed distribution, while categorical variables were depicted as frequency or percentage. For assessing statistical variations between group means and proportions, we utilized one-way ANOVA (for normal distribution), Kruskal–Wallis H test (for skewed distribution), and chi-square tests (for categorical variables).

We employed two separate multivariable weighted multinomial logistic regression models to evaluate the relationship between sleep duration and varying levels of estradiol, categorizing them as high and low relative to normal levels. Additionally, we conducted stratified analyses based on gender and age groups, with adult women further categorized by menopausal status. Three models were applied: the nonadjusted model, which did not include any covariates; the minimally adjusted model, which controlled for age and race; and the fully adjusted model, which accounted for multiple factors including age, BMI, race, alcohol consumption, hypertension, diabetes, smoking status, testosterone levels, SHBG levels, educational attainment, time of examination, and physical activity. These models were designed to explore associations between sleep duration and serum estradiol, rather than to infer causal relationships. Given the cross-sectional nature of the NHANES data, the causal direction of any observed associations cannot be determined. Therefore, we selected an analytical approach ideal for identifying correlations in cross-sectional data. We analyzed the data using R software (Version 4.4.1) and EmpowerStats (https://www.empowerstats.com). We considered a *p* value of < 0.05 as statistically significant [11].

## 3. Results

Table 1 shows the distribution of selected characteristics stratified by sleep duration. Participants with the longest sleep duration (≥ 9 h) were generally older, with a mean age of 52.12 years, and had a higher percentage of women (36.70%), particularly postmenopausal women (20.91%). This group also had a higher prevalence of diabetes (25.90%). In contrast, those with the shortest sleep duration (≤ 6 h) were more likely to be male (77.15%), non-Hispanic Black (24.93%), and current smokers (24.86%), with a higher mean BMI (29.63 kg/m^2^). Estradiol levels varied slightly across the sleep duration groups. The median estradiol levels were 23.10 pg/mL for the 6–9-h group, 23.65 pg/mL for the ≤ 6-h group, and 22.90 pg/mL for the ≥ 9-h group. Most participants had normal estradiol levels regardless of sleep duration. Furthermore, considering the sampling weights, we conducted a weighted analysis of the study population characteristics, with detailed information provided in Table S2.

### 3.1. Male Population

The results in Table 2 indicate that there is no significant association between sleep duration and serum estradiol concentrations among American men. In the overall population, compared to the reference group (6–9 h of sleep), the odds ratios (ORs) for low estradiol concentrations (≤ 10 pg/mL) and high estradiol concentrations (≥ 50 pg/mL) for the shorter sleep duration group (≤ 6 h) were 0.85 (95% CI: 0.45, 1.61) and 1.07 (95% CI: 0.61, 1.88), respectively, neither of which reached statistical significance. For the longer sleep duration group (≥ 9 h), the ORs for low and high estradiol concentrations were 0.84 (95% CI: 0.38, 1.84) and 1.02 (95% CI: 0.49, 2.14), also not statistically significant. This suggests that variations in serum estradiol concentrations among men do not significantly differ with varying sleep durations.

In different age groups (20–40, 41–64, ≥ 65 years), the association between sleep duration and estradiol concentrations remained nonsignificant. For instance, in the 20–40 age group, the OR for low estradiol concentrations in the ≤ 6-h sleep group was 0.59 (95% CI: 0.17, 2.09), and for high estradiol, it was 1.44 (95% CI: 0.51, 4.05), both not statistically significant. In the 41–64 and ≥ 65 age groups, the ORs for low or high estradiol concentrations across different sleep duration groups (≤ 6 and ≥ 9 h) included 1 within their 95% confidence intervals, indicating no significant differences.

### 3.2. Female Population

The results in Table 3 show no significant association between sleep duration and serum estradiol concentrations among American adult women. In the overall population, the ORs for low estradiol concentrations in the ≤ 6- and ≥ 9-h sleep groups compared to the 6–9-h sleep group were 1.32 (95% CI: 0.65, 2.70) and 1.30 (95% CI: 0.60, 2.81), respectively, indicating no significant changes in estradiol levels. For high estradiol concentrations, the OR for the ≤ 6-h sleep group was 1.10 (95% CI: 0.58, 2.12), and for the ≥ 9-h sleep group, it was 0.67 (95% CI: 0.32, 1.38), also showing no significant changes.

In both premenopausal and postmenopausal groups, the association between sleep duration and estradiol concentrations remained nonsignificant. For postmenopausal women, given that the normal estradiol range is ≤ 20 pg/mL, only a high estradiol group exists. The results indicated that in the high estradiol concentration group (> 20 pg/mL), the OR for the ≤ 6-h sleep group was 0.98 (95% CI: 0.42, 2.29), while for the ≥ 9-h sleep group, it was 0.80 (95% CI: 0.30, 2.13), with no significant changes in estradiol concentrations.

## 4. Discussion

Our study examined the association between sleep duration and serum estradiol concentrations among adult American men and women, utilizing data from the NHANES 2013–2016 cycles. Our analysis revealed no significant association between sleep duration and serum estradiol concentrations across different age groups and sexes. Specifically, the ORs for both low and high estradiol concentrations remained statistically nonsignificant when comparing sleep durations of ≤ 6 and ≥ 9 h to the reference group of 6–9 h in fully adjusted models.

Early surveys have shown that females report more sleep-related issues than males, including difficulties falling asleep, excessive daytime sleepiness, trouble maintaining sleep, and not feeling refreshed upon waking [17]. Adult women experience significant hormonal fluctuations in estrogen levels during the menstrual cycle, pregnancy, perimenopause, and postmenopause. During the early follicular phase of the menstrual cycle, both progesterone and estrogen secretion decrease, which relieves the negative feedback inhibition on the hypothalamus and pituitary gland, leading to an increased secretion of FSH and LH, especially FSH. This causes a group of follicles to be recruited into rapid growth, and estrogen secretion increases. During the mid-cycle, as the dominant follicle matures, estrogen secretion increases further. After ovulation, the ovaries enter the luteal phase, where estrogen secretion temporarily decreases. However, with the action of LH, the corpus luteum develops, and both progesterone and estrogen secretion increase, with a more notable increase in progesterone. A second peak in estrogen and progesterone secretion usually occurs 7–8 days after ovulation. If fertilization does not occur, the corpus luteum begins to regress on the 9th to 10th day after ovulation, leading to a decrease in both estrogen and progesterone levels. During pregnancy, estrogen levels gradually increase, with estriol levels reaching up to 1,000 times higher than in nonpregnant women by the end of pregnancy. During the perimenopausal period, ovarian function begins to decline, and the ovaries' response to FSH and LH diminishes. Follicles often become arrested at various stages of development and fail to ovulate, resulting in reduced estrogen secretion. After menopause, estrogen levels remain low for an extended period. In contrast, adult males exhibit minimal fluctuations in estradiol levels, with the NHANES data indicating a normal range of 10–50 pg/mL. Given the significant hormonal differences between adult males and females, we have stratified the analysis by gender.

Our analysis of the NHANES 2013–2016 dataset revealed no significant association between sleep duration and serum estradiol concentrations among premenopausal and postmenopausal women, a finding supported by the previous literature. For instance, a cross-sectional laboratory study of 33 perimenopausal women aged 43–52 years found no relationship between sleep patterns and hormone levels [including estradiol and follicle-stimulating hormone (FSH)] in individuals diagnosed with insomnia [18]. Similarly, total sleep duration among both perimenopausal and postmenopausal women was not correlated with symptoms, hormone levels (such as FSH and 17A-estradiol), age, or lifestyle factors [19]. Additionally, studies comparing women with and without postpartum insomnia [20] and those with paradoxical and psychophysiological insomnia (*n* = 36) to normal sleepers (*n* = 17) found no significant differences in estradiol levels [21]. A study indicated that poor sleep in middle-aged women is not inherently associated with menopause [22]. Another study of regularly menstruating women aged 24–36 in Poland showed no statistically significant relationship between sleep duration and estradiol levels [23]. However, some research findings diverge from our results. The BioCycle Study reported that each additional hour of daily sleep was associated with a 3.9% increase in mean estradiol concentrations [6]. Conversely, a study of pregnant Japanese women found that maternal estradiol levels during the 29th week were inversely related to sleep duration on weekends [7]. Furthermore, an analysis involving 45 perimenopausal women revealed that a higher frequency of awakenings correlated with lower estradiol levels [24]. Additionally, lower estradiol and elevated luteinizing hormone (LH) levels were significantly associated with poor sleep quality indices [25]. Despite these inconsistencies, several studies have demonstrated that estrogen replacement therapy can improve sleep quality in postmenopausal women [26–28]. Moreover, a recent meta-analysis revealed that the combination of estrogen and progesterone had a positive effect on sleep disturbances, whereas estrogen monotherapy did not [29]. These varied findings emphasize the complexity of the relationship between sleep and estradiol levels, underscoring the need for further research specifically focused on adult females.

Relative to women, there are fewer studies examining the relationship between estradiol and sleep in men. Our findings indicate no significant association between sleep duration and serum estradiol concentrations among American men. This aligns with the study by Goh and Tong, who found no relationship between sleep duration and estradiol levels in a cohort of 531 Singaporean Chinese men aged 29–72 years [8]. Similarly, Iranzo et al. reported no differences in serum estradiol levels between 14 male idiopathic rapid eye movement (REM) sleep behavior disorder (RBD) patients and 16 healthy matched controls [30]. These results suggest that sleep behavior in men might be less sensitive to the suppressive effects of gonadal steroids. This hypothesis is supported by the preclinical study of Cusmano et al., which found that the magnitude of change in sleep behavior induced by either estradiol or testosterone was greater in female rats compared to male rats [31]. However, in younger populations, the relationship may differ. Hazell et al. investigated 158 male participants aged 10–12 years living in rural Australia and discovered that higher testosterone and estradiol levels were associated with poorer sleep [9]. Moreover, the Wibowo et al. study indicates that estradiol treatment in castrated male rats promotes baseline wakefulness during the active phase and facilitates the recovery of REM sleep after acute sleep loss. This suggests a potential benefit of estradiol treatment for improving sleep quality in androgen-deprived men, though this remains to be further investigated [32]. The variability in these findings highlights the complexity of the relationship between sleep and estradiol in men. While some studies suggest a potential link, our research indicates that sleep duration alone may not be a significant factor in determining estradiol levels. These differences underscore the need for continued research into how various factors, including genetic predispositions, lifestyle habits, and environmental influences, may modulate the relationship between sleep and hormone levels.

The main reasons for the lack of positive findings in our study are as follows: First, missing information on the timing of blood draws during the menstrual cycle: NHANES data do not include information about the timing of blood draws relative to the participants' menstrual cycle. As is well known, estradiol levels in women vary depending on the phase of the menstrual cycle (e.g., menstruation, early follicular phase, late follicular phase, mid-cycle peak, and luteal phase). However, NHANES data do not specify which phase the adult women were in at the time of blood sample collection. Additionally, previous studies in the literature have been inconsistent regarding the reporting of menstrual cycle timing for hormone collection. For example, one study explored the relationship between sleep changes during the perimenopausal and postmenopausal periods and emotional, hormonal, and lifestyle factors but did not provide detailed information on the specific menstrual cycle timing for hormone collection [19]. Another study aimed at determining the association between female reproductive hormones and perimenopausal sleep discontinuity independent of nocturnal vasomotor symptoms (nVMS) and depressive symptoms (DepSx); serum reproductive hormones (estradiol, FSH, and progesterone) were assessed weekly over an 8-week observational period [24]. However, many studies that recorded the timing of hormone collection chose to collect estradiol during the early follicular phase [18, 33], which aligns with our clinical practice. Therefore, we assumed that all NHANES adult female samples in our study were collected during the early follicular phase. Second, differences in the grouping criteria of NHANES data: The reference range and grouping criteria for estradiol in NHANES differ from those in other studies. Although a population-based reference range for estradiol has not yet been universally established, the NHANES Laboratory Procedures Manual provides the normal reference range for estradiol (Table S1). We assumed that estradiol samples from adult women in NHANES were collected during the follicular phase, and thus, we defined the normal reference range for estradiol in adult women as 20–350 pg/mL [12] while categorizing postmenopausal women and men according to the reference ranges provided by NHANES. However, previous studies investigating the relationship between estradiol and sleep duration did not always group estradiol levels [18, 19], or the grouping criteria were inconsistent. For example, one study defined normal estradiol levels in postmenopausal women as below 15 pg/mL, whereas NHANES defines it as ≤ 20 pg/mL [24]. Third, small sample size in the estradiol deficiency and excess groups: The number of participants in the estradiol deficiency and excessive estradiol groups was very small, making it difficult to obtain significant results. As shown in Table 1, approximately 95% of the study population had normal estradiol levels, while the estradiol deficiency and excessive estradiol groups accounted for less than 3% each. In many other NHANES-based studies investigating associations with sex hormones, researchers often use tertiles [10], quartiles [34], or custom groupings [10, 35], which increases the number of individuals in the low or high estradiol groups, thereby increasing the likelihood of positive findings. For example, in the study by Hernández-Pérez JG et al. on the relationship between sleep duration and serum testosterone levels in men and women [10], total testosterone (Total T) was categorized into low, moderate, and high groups. For men, the cutoff values were low < 300 ng/dL, moderate = 300–850 ng/dL (reference group), and high > 850 ng/dL; for women, tertile distribution was used, with low ≤ 15.06 ng/dL, moderate = 15.07–24.4 ng/dL (reference group), and high > 24.4 ng/dL. It is evident that the normal testosterone range for men in Hernández-Pérez JG's study is much lower than the NHANES-defined normal range (280–1100 ng/dL), and the use of tertile distribution for women allowed more individuals to be categorized into low or high testosterone groups, thus increasing the likelihood of detecting positive results. In contrast, our stricter grouping criteria made it more difficult to obtain positive findings. In the future, if NHANES could collect longitudinal data on sex hormones over one or two menstrual cycles in adult women (e.g., including visits on the second day of menstruation, mid- and late follicular phases, LH peak day, predicted ovulation day, and early, mid-, and late luteal phases, totaling eight visits) [6], we would be able to group individuals more precisely based on estradiol levels at different stages of the menstrual cycle. This approach would provide more scientifically robust results that better reflect the characteristics of the population.

In our study, we aimed to observe the association between sleep duration and serum estradiol concentrations. It is important to consider the potential for reverse causality in this relationship. While our analysis did not reveal significant associations, it remains plausible that variations in estradiol concentrations could affect sleep patterns. For instance, fluctuations in hormone levels, particularly during different life stages, may lead to alterations in sleep quality and duration. This bidirectional relationship warrants further investigation to clarify the causal pathways involved.

Additionally, we recognize the possibility of residual confounding in our analysis. Despite controlling for several known confounders such as age, BMI, and lifestyle factors, there may be unmeasured variables that could influence both sleep and estradiol levels. Factors such as psychological well-being, stress levels, and environmental conditions may play significant roles in this relationship. Future research should aim to identify and control for these potential confounders to provide a more comprehensive understanding of the interplay between sleep duration and hormonal regulation.

Our study has several strengths. Firstly, our data source is the NHANES database, which ensures the standardization of sleep questionnaire surveys and the accuracy of serum hormone measurements. Additionally, we applied weighting to the data, ensuring that the final results are representative of the overall health status of American adults. More importantly, our study conducted stratified analyses by age and gender, providing valuable references for clinical research. However, there are several limitations to our study. First, the data on sleep duration in our study were obtained through a single in-home interview using a CAPI system. This system records sleep duration in half-hour increments, which may not accurately reflect the respondents' actual sleep duration due to potential reporting errors and recall bias. Additionally, the NHANES questionnaire does not provide information on other aspects of sleep, such as sleep latency, habitual sleep efficiency, use of sleep medications, and daytime dysfunction, limiting our ability to assess how these factors might influence sleep duration. To obtain a more comprehensive understanding of sleep duration and its relationship with estradiol levels, future studies could utilize more sophisticated sleep assessment tools, such as the Pittsburgh Sleep Quality Index, or employ polysomnography (PSG) [36] and convenient home sleep monitoring devices, including smart wristbands. Furthermore, we acknowledge that sleep duration is a complex concept influenced by various factors, including occupational type (e.g., night shifts), work-related stress, the day of the week (i.e., weekdays vs. weekends) [37], and napping habits [38]. Unfortunately, NHANES data do not capture these variables either [39]. Furthermore, the study lacked data on gonadotropin-releasing hormone (GnRH), gonadotropins, and key enzymes involved in hormone response, limiting our ability to explore potential biological mechanisms further. It is well established that there is feedback regulation between the hypothalamus, pituitary, and gonads. The hypothalamus secretes GnRH, which stimulates the pituitary to release gonadotropins (LH and FSH), subsequently promoting the gonads (testes and ovaries) to secrete sex hormones (testosterone from the testes, and estradiol and progesterone from the ovaries). Previous studies have confirmed the complex relationship between sleep and the HPG axis. For instance, studies have shown a positive association between endogenous progesterone and sleep, with progesterone promoting sleep in both younger and older women [40]. Furthermore, declining or low progesterone levels during the menopausal transition have been linked to sleep disturbances, such as reduced sleep efficiency and shorter sleep duration [41]. A longitudinal study on the menopausal transition in women revealed that a more rapid increase in FSH was associated with longer sleep duration but poorer sleep quality [42]. Additionally, a sleep deprivation experiment in male rats showed that while sleep deprivation did not negatively impact hypothalamic GnRH secretion, it did have a negative effect on the pituitary, particularly LH secretion [43]. However, it is important to note that NHANES data only assessed hormone serum levels at a single time point and did not include hormones relevant to the HPG axis. As a result, it fails to capture the full range of changes in the estrogen-related HPG axis rhythm, limiting our ability to further explore the potential biological mechanisms underlying the relationship between sleep and HPG axis hormones. It is important to note that this study is cross-sectional and thus cannot establish causality. Although we did not find significant associations between sleep duration and serum estradiol concentrations, the possibility of an underlying causal relationship cannot be ruled out. This cross-sectional design captures data at a single point in time, limiting our ability to track changes in variables over time or to ascertain causal sequences. Future research should employ longitudinal designs to overcome these limitations and validate our findings [11].

## 5. Conclusions

In conclusion, our study found no significant association between sleep duration and serum estradiol concentrations among adult American men and women. These findings suggest that sleep duration alone may not be a critical factor influencing estradiol levels in the general population. However, it is important to note that the cross-sectional nature of our study limits our ability to draw causal inferences regarding the relationship between sleep duration and estradiol concentrations. Future research should move beyond single time-point serum hormone collections to include one to two menstrual cycles in women. Convenient home sleep monitoring devices, such as smartphone apps and smartwatches, can be employed for longitudinal sleep assessments. Additionally, we recommend the dynamic collection of hormones related to the HPG axis, including estradiol, along with long-term follow-up across different age groups. This approach will facilitate a more comprehensive understanding of the relationship between sleep duration and estradiol levels.

## Figures and Tables

**Table 1:** Characteristics of US adults aged ≥ 20 years by sleep duration in the NHANES 2013–2016 total sample (*n* = 5406).

Characteristics	6–9 h (*n* = 3101)	≤ 6 h (*n* = 1444)	≥ 9 h (*n* = 861)	*p* value
Age (year, mean ± SD)	49.12 (17.88)	48.22 (16.73)	52.12 (20.55)	< 0.001
BMI (kg/m^2^, mean ± SD)	28.66 (6.37)	29.63 (7.00)	28.85 (6.45)	< 0.001
Testosterone (ng/dL, median, Q1–Q3)	328.00 (72.60–461.00)	336.00 (153.50–469.00)	268.00 (27.00–462.00)	< 0.001
SHBG (nmol/l, median, Q1–Q3)	41.29 (28.66–61.85)	40.11 (28.29–58.18)	49.14 (32.95–72.19)	< 0.001
Estradiol (pg/mL, median, Q1–Q3)	23.10 (16.50–30.90)	23.65 (16.70–31.30)	22.90 (13.80–33.00)	0.040
Normal estradiol (*N*, %)	2950 (95.13%)	1368 (94.74%)	811 (94.19%)	
Estradiol deficiency (*N*, %)	73 (2.35%)	33 (2.29%)	25 (2.90%)	
Excessive estradiol (*N*, %)	78 (2.52%)	43 (2.98%)	25 (2.90%)	
Gender				< 0.001
Male (*N*, %)	2324 (74.94%)	1114 (77.15%)	545 (63.30%)	
20–40 male (*N*, %)	871 (28.09%)	439 (30.40%)	191 (22.18%)	
41–64 male (*N*, %)	963 (31.05%)	493 (34.14%)	178 (20.67%)	
≥ 65 male (*N*, %)	490 (15.80%)	182 (12.60%)	176 (20.44%)	
Female (*N*, %)	777 (25.06%)	330 (22.85%)	316 (36.70%)	
Premenopausal female (*N*, %)	368 (11.87%)	145 (10.04%)	136 (15.80%)	
Postmenopausal female (*N*, %)	409 (13.19%)	185 (12.81%)	180 (20.91%)	
Race				< 0.001
Mexican American (*N*, %)	563 (18.16%)	220 (15.24%)	170 (19.74%)	
Other Hispanic (*N*, %)	337 (10.87%)	182 (12.60%)	111 (12.89%)	
Non-Hispanic white (*N*, %)	1174 (37.86%)	444 (30.75%)	324 (37.63%)	
Non-Hispanic black (*N*, %)	481 (15.51%)	360 (24.93%)	147 (17.07%)	
Non-Hispanic Asian (*N*, %)	455 (14.67%)	182 (12.60%)	83 (9.64%)	
Other race (*N*, %)	91 (2.93%)	56 (3.88%)	26 (3.02%)	
Education level				< 0.001
Less than 9th grade (*N*, %)	343 (11.06%)	135 (9.35%)	161 (18.70%)	
9–11th grade (*N*, %)	406 (13.09%)	212 (14.68%)	128 (14.87%)	
High school graduate	655 (21.12%)	371 (25.69%)	224 (26.02%)	
Some college or AA degree (*N*, %)	839 (27.06%)	450 (31.16%)	204 (23.69%)	
College graduate or above (*N*, %)	858 (27.67%)	276 (19.11%)	144 (16.72%)	
Diabetes				< 0.001
No (*N*, %)	2552 (82.30%)	1169 (80.96%)	638 (74.10%)	
Yes (*N*, %)	549 (17.70%)	275 (19.04%)	223 (25.90%)	
Examine time				0.752
Morning (*N*, %)	1484 (47.86%)	680 (47.09%)	407 (47.27%)	
Afternoon (*N*, %)	1155 (37.25%)	540 (37.40%)	336 (39.02%)	
Evening (*N*, %)	462 (14.90%)	224 (15.51%)	118 (13.70%)	
Hypertension				< 0.001
No (*N*, %)	1878 (60.56%)	808 (55.96%)	453 (52.61%)	
Yes (*N*, %)	1223 (39.44%)	636 (44.04%)	408 (47.39%)	
Physical activity				0.988
Nonactivity (*N*, %)	1704 (54.95%)	785 (54.36%)	459 (53.31%)	
0.1–0.9 h/month (*N*, %)	277 (8.93%)	135 (9.35%)	80 (9.29%)	
1.0–3.4 h/month (*N*, %)	558 (17.99%)	264 (18.28%)	161 (18.70%)	
≥ 6 h/month (*N*, %)	562 (18.12%)	260 (18.01%)	161 (18.70%)	
Alcohol intake				0.001
Nondrinker (*N*, %)	840 (27.09%)	387 (26.80%)	285 (33.10%)	
1–5 drinks/month (*N*, %)	1524 (49.15%)	764 (52.91%)	406 (47.15%)	
5–10 drinks/month (*N*, %)	258 (8.32%)	99 (6.86%)	59 (6.85%)	
10+ drinks/month (*N*, %)	479 (15.45%)	194 (13.43%)	111 (12.89%)	
Smoking status				< 0.001
Nonsmoker (*N*, %)	1749 (56.40%)	725 (50.21%)	490 (56.91%)	
Former smoker (*N*, %)	796 (25.67%)	360 (24.93%)	201 (23.34%)	
Current smoker (*N*, %)	556 (17.93%)	359 (24.86%)	170 (19.74%)	

Abbreviations: BMI, body mass index; SHBG, sex hormone–binding globulin.

**Table 2:** Multivariable multinomial logistic regression of the association between sleep duration and estradiol categories in US adult males (NHANES 2013–2016).

Age groups/sleep duration (h)	Estradiol concentrations (pg/mL) categories^a^					
	Low ≤ 10, OR (95% CI)			High ≥ 50, OR (95% CI)		
	Nonadjusted model^b^	Minimally adjusted model^c^	Fully adjusted model^d^	Nonadjusted model^b^	Minimally adjusted model^c^	Fully adjusted model^d^
All						
6–9 h (*n* = 1874)	Ref	Ref	Ref	Ref	Ref	Ref
≤ 6 h (*n* = 1114)	0.83 (0.47, 1.45)	0.88 (0.50, 1.55)	0.85 (0.45, 1.61)	1.21 (0.74, 2.00)	1.14 (0.69, 1.90)	1.07 (0.61, 1.88)
≥ 9 h (*n* = 545)	1.10 (0.56, 2.15)	1.04 (0.53, 2.04)	0.84 (0.38, 1.84)	1.30 (0.69, 2.43)	1.24 (0.66, 2.33)	1.02 (0.49, 2.14)
20–40 years old						
6–9 h (*n* = 871)	Ref	Ref	Ref	Ref	Ref	Ref
≤ 6 h (*n* = 439)	0.44 (0.15, 1.31)	0.49 (0.16, 1.47)	0.59 (0.17, 2.09)	1.65 (0.71, 3.85)	1.43 (0.60, 3.41)	1.44 (0.51, 4.05)
≥ 9 h (*n* = 191)	0.77 (0.22, 2.63)	0.82 (0.24, 2.84)	0.71 (0.13, 3.75)	1.91 (0.67, 5.50)	1.63 (0.56, 4.76)	1.02 (0.27, 3.78)
41–64 years old						
6–9 h (*n* = 963)	Ref	Ref	Ref	Ref	Ref	Ref
≤ 6 h (*n* = 493)	0.83 (0.32, 2.18)	0.85 (0.32, 2.23)	0.61 (0.20, 1.91)	0.79 (0.36, 1.74)	0.75 (0.34, 1.65)	0.60 (0.23, 1.57)
≥ 9 h (*n* = 178)	1.55 (0.50, 4.76)	1.51 (0.49, 4.68)	1.55 (0.43, 5.69)	0.74 (0.22, 2.50)	0.68 (0.20, 2.32)	0.58 (0.13, 2.64)
≥ 65 years old						
6–9 h (*n* = 490)	Ref	Ref	Ref	Ref	Ref	Ref
≤ 6 h (*n* = 182)	1.77 (0.68, 4.64)	1.67 (0.62, 4.50)	2.43 (0.56, 10.64)	1.85 (0.65, 5.29)	2.08 (0.72, 6.04)	2.35 (0.61, 8.97)
≥ 9 h (*n* = 176)	1.02 (0.32, 3.26)	0.89 (0.28, 2.89)	0.61 (0.12, 3.07)	1.56 (0.52, 4.73)	1.61 (0.52, 4.94)	1.73 (0.38, 7.83)

*Note:* All models were weighted.

Abbreviations: CI, confidence interval; OR, odds ratio; Ref, reference group.

^a^Reference outcome for the multinomial analysis is the normal ranges of estradiol levels.

^b^Nonadjusted model: no covariates were adjusted.

^c^Minimally adjusted model: adjusted for age and race.

^d^Fully adjusted model: For the total population, adjustments were made for age, body mass index, race, alcohol intake, hypertension, diabetes, smoking status, testosterone level, sex hormone-binding globulin (SHBG) level, education level, examine time, and physical activity. For age-stratified populations, the model was adjusted for all the aforementioned variables except age.

**Table 3:** Multivariable multinomial logistic regression of the association between sleep duration and estradiol categories in US adult females (NHANES 2013–2016).

Age groups/sleep duration (h)	Estradiol concentrations (pg/mL) categories^a^					
	Low ≤ 20, OR (95% CI)			High (premenopausal ≥ 350, postmenopausal > 20), OR (95% CI)		
	Nonadjusted model^b^	Minimally adjusted model^c^	Fully adjusted model^d^	Nonadjusted model^b^	Minimally adjusted model^c^	Fully adjusted model^d^
All						
6–9 h (*n* = 777)	Ref	Ref	Ref	Ref	Ref	Ref
≤ 6 h (*n* = 330)	1.28 (0.69, 2.39)	1.27 (0.67, 2.41)	1.32 (0.65, 2.70)	1.24 (0.69, 2.22)	1.19 (0.66, 2.14)	1.10 (0.58, 2.12)
≥ 9 h (*n* = 316)	1.15 (0.60, 2.19)	1.17 (0.60, 2.28)	1.30 (0.60, 2.81)	0.84 (0.43, 1.64)	0.77 (0.39, 1.50)	0.67 (0.32, 1.38)
Premenopausal						
6–9 h (*n* = 368)	Ref	Ref	Ref	Ref	Ref	Ref
≤ 6 h (*n* = 145)	1.40 (0.74, 2.65)	1.54 (0.79, 3.02)	1.39 (0.63, 3.07)	1.05 (0.20, 5.48)	0.85 (0.15, 4.66)	0.66 (0.05, 8.99)
≥ 9 h (*n* = 136)	1.31 (0.67, 2.55)	1.82 (0.90, 3.68)	1.95 (0.82, 4.60)	1.68 (0.40, 7.13)	1.62 (0.37, 7.16)	1.26 (0.19, 8.42)
Postmenopausal						
6–9 h (*n* = 409)	—	—	—	Ref	Ref	Ref
≤ 6 h (*n* = 185)	—	—	—	1.20 (0.63, 2.25)	1.05 (0.52, 2.11)	0.98 (0.42, 2.29)
≥ 9 h (*n* = 180)	—	—	—	0.66 (0.31, 1.43)	0.90 (0.40, 2.04)	0.80 (0.30, 2.13)

*Note:* All models were weighted.

Abbreviations: CI, confidence interval; OR, odds ratio; Ref, reference group.

^a^Reference outcome for the multinomial analysis is the normal ranges of estradiol levels.

^b^Nonadjusted model: no covariates were adjusted.

^c^Minimally adjusted model: adjusted for age and race.

^d^Fully adjusted model: adjusted for age, body mass index, race, alcohol intake, hypertension, diabetes, smoking status, testosterone level, sex hormone-binding globulin (SHBG) level, education level, examine time, and physical activity.

## Data Availability

All data generated or analyzed in this study were provided by the National Center for Health Statistics of the US Centers for Disease Control and Prevention (https://www.cdc.gov/nchs/nhanes/index.htm). For further data inquiries, please contact the corresponding author of this manuscript (Xianying Zhou).

## Supporting Information

Table S1: Normal ranges of total estradiol and total testosterone in serum from the NHANES Laboratory Procedures Manual. Table S2: Weighted characteristics of US adults aged ≥ 20 years by sleep duration in the NHANES 2013–2016 total sample (*n* = 5406).
