# Supplementary material for: Association of Sleep Duration With Serum Estradiol Concentrations Among American Men and Women: Evidence From NHANES 2013–2016
Source: Int J Endocrinol. 2025 Feb 7;2025:7863420. doi: 10.1155/ije/7863420 (PMC11828656; doi:10.1155/ije/7863420)
Supplement: Supporting Information — Additional supporting information can be found online in the Supporting Information section. [file 7863420.f1.docx]

**Table S1: Normal ranges of total estradiol and total testosterone in serum from the NHANES Laboratory Procedures Manual**

| Analyte-Sex | Group | Value Range | |
| --- | --- | --- | --- |
|  |  | pg/ml | pmol/L |
| Total Estradiol-Male | Adult | 10-50 | 37-184 |
| Total Estradiol-Female | Early follicular phase | 20-150 | 73-551 |
|  | Late follicular phase | 40-350 | 147-1285 |
|  | Midcycle peak | 150-750 | 551-2753 |
|  | Luteal phase | 30-450 | 110-1652 |
|  | Postmenopausal | ≤20 | ≤73 |
| Total Testosterone | Group | Value Range | |
|  |  | ng/dL | nmol/L |
| Male | Adult | 280-1100 | 9.71-34.7 |
| Female | Adult | 15-70 | 0.52-2.43 |

**Table S2: Weighted Characteristics of US Adults Aged ≥ 20 Years by Sleep Duration in the NHANES 2013–2016 Total Sample (n = 5406)**

| Characteristics | 6-9 hrs (n=3101)  Weighted % (95% CI) | ≤6 hrs (n=1444)  Weighted % (95% CI) | ≥9 hrs (n=861)  Weighted % (95% CI) | P-value |
| --- | --- | --- | --- | --- |
| Age | 47.22 (46.19 ,48.25) | 46.00 (44.83 ,47.18) | 49.27 (47.05 ,51.48) | 0.0522 |
| BMI | 28.90 (28.57 ,29.24) | 29.71 (29.28 ,30.15) | 28.77 (28.17 ,29.37) | 0.0015 |
| Testosterone | 344.18 (331.68 ,356.67) | 345.14 (333.05 ,357.22) | 303.42 (286.97 ,319.88) | 0.0003 |
| SHBG | 47.28 (45.38 ,49.18) | 46.92 (45.08 ,48.75) | 56.48 (53.43 ,59.54) | <0.0001 |
| Estradiol | 30.04 (28.53 ,31.56) | 31.24 (28.56 ,33.91) | 34.35 (30.45 ,38.25) | 0.1285 |
| Normol Estradiol | 95.42 (93.87 ,96.60) | 94.94 (93.42 ,96.13) | 95.07 (93.21 ,96.44) |  |
| Estradiol deficiency | 2.14 (1.49 ,3.07) | 2.22 (1.56 ,3.14) | 2.31 (1.42 ,3.74) |  |
| Excessive Estradiol | 2.43 (1.68 ,3.52) | 2.84 (1.89 ,4.25) | 2.62 (1.65 ,4.16) |  |
| Gender |  |  |  | <0.0001 |
| Male | 80.93 (78.59 ,83.08) | 80.13 (77.83 ,82.24) | 68.71 (65.48 ,71.76) |  |
| 20-40 male | 32.44 (30.22 ,34.75) | 33.84 (30.65 ,37.18) | 27.96 (23.24 ,33.22) |  |
| 41-64 male | 35.12 (32.92 ,37.38) | 37.23 (33.88 ,40.71) | 22.67 (19.21 ,26.55) |  |
| ≥ 65 male | 13.38 (11.80 ,15.13) | 9.06 (7.34 ,11.14) | 18.08 (13.94 ,23.12) |  |
| Female | 19.07 (16.92 ,21.41) | 19.87 (17.76 ,22.17) | 31.29 (28.24 ,34.52) |  |
| Premenopausal female | 9.34 (7.85 ,11.09) | 9.86 (8.20 ,11.83) | 15.19 (12.69 ,18.06) |  |
| Postmenopausal female | 9.72 (8.33 ,11.31) | 10.01 (8.31 ,12.01) | 16.11 (13.29 ,19.39) |  |
| Race |  |  |  | <0.0001 |
| Mexican American | 10.41 (7.11 ,15.00) | 11.75 (8.68 ,15.71) | 11.15 (6.98 ,17.36) |  |
| Other Hispanic | 5.95 (4.41 ,7.98) | 8.30 (6.35 ,10.79) | 7.17 (5.00 ,10.19) |  |
| Non-Hispanic White | 66.04 (60.12 ,71.50) | 54.20 (48.17 ,60.10) | 63.32 (56.09 ,69.99) |  |
| Non-Hispanic Black | 7.76 (6.10 ,9.84) | 15.72 (12.79 ,19.17) | 10.41 (7.27 ,14.68) |  |
| Non-Hispanic Asian | 6.85 (5.14 ,9.09) | 6.40 (4.64 ,8.77) | 5.41 (3.49 ,8.29) |  |
| Other Race | 2.98 (2.17 ,4.08) | 3.63 (2.62 ,5.00) | 2.54 (1.56 ,4.11) |  |
| EducationLevel |  |  |  | <0.0001 |
| Less than 9th grade | 5.88 (4.41 ,7.80) | 5.69 (4.66 ,6.92) | 9.90 (6.95 ,13.90) |  |
| 9-11th grade | 9.78 (8.01 ,11.89) | 11.66 (9.83 ,13.77) | 12.00 (9.56 ,14.97) |  |
| High school graduate | 20.14 (17.86 ,22.63) | 26.25 (22.14 ,30.83) | 28.41 (24.93 ,32.16) |  |
| Some college or AA degree | 29.51 (26.30 ,32.94) | 34.18 (30.86 ,37.67) | 28.53 (25.11 ,32.21) |  |
| College graduate or above | 34.70 (29.75 ,40.00) | 22.22 (17.94 ,27.18) | 21.16 (16.02 ,27.42) |  |
| Diabetes |  |  |  | 0.0110 |
| No | 86.02 (84.07 ,87.76) | 84.60 (81.77 ,87.06) | 80.60 (75.68 ,84.72) |  |
| Yes | 13.98 (12.24 ,15.93) | 15.40 (12.94 ,18.23) | 19.40 (15.28 ,24.32) |  |
| Examine Time |  |  |  | 0.7674 |
| Morning | 48.55 (46.11 ,51.00) | 47.44 (44.17 ,50.74) | 49.01 (41.74 ,56.31) |  |
| Afternoon | 35.44 (33.02 ,37.94) | 36.68 (33.67 ,39.80) | 37.64 (31.25 ,44.50) |  |
| Evening | 16.00 (14.09 ,18.12) | 15.88 (12.99 ,19.27) | 13.35 (10.62 ,16.64) |  |
| Hypertension |  |  |  | 0.0505 |
| No | 63.43 (61.24 ,65.56) | 58.57 (54.82 ,62.23) | 58.96 (53.56 ,64.16) |  |
| Yes | 36.57 (34.44 ,38.76) | 41.43 (37.77 ,45.18) | 41.04 (35.84 ,46.44) |  |
| PhysicalActivity |  |  |  | 0.6651 |
| Non-activity | 55.12 (52.34 ,57.87) | 57.71 (54.03 ,61.30) | 54.33 (50.02 ,58.58) |  |
| 0.1-0.9 hrs/month | 8.58 (7.38 ,9.94) | 9.22 (7.90 ,10.73) | 8.20 (5.88 ,11.31) |  |
| 1.0-3.4 hrs/month | 18.98 (17.39 ,20.67) | 17.19 (14.63 ,20.08) | 19.91 (15.48 ,25.24) |  |
| ≥6 hrs/month | 17.32 (15.71 ,19.06) | 15.89 (13.87 ,18.14) | 17.56 (15.13 ,20.28) |  |
| AlcoholIntake |  |  |  | <0.0001 |
| Non-drinker | 20.05 (17.29 ,23.14) | 22.50 (18.93 ,26.52) | 27.83 (23.98 ,32.04) |  |
| 1-5 drinks/month | 48.67 (45.26 ,52.09) | 53.17 (48.98 ,57.31) | 48.23 (44.13 ,52.35) |  |
| 5-10 drinks/month | 10.39 (9.19 ,11.73) | 7.91 (6.08 ,10.23) | 7.06 (5.15 ,9.61) |  |
| 10+ drinks/month | 20.89 (18.50 ,23.51) | 16.42 (13.10 ,20.38) | 16.88 (13.68 ,20.64) |  |
| SmokingStatus |  |  |  | <0.0001 |
| Non-smoker | 56.85 (54.70 ,58.98) | 48.20 (43.82 ,52.62) | 55.31 (51.60 ,58.97) |  |
| Former smoker | 26.92 (24.70 ,29.27) | 25.81 (22.73 ,29.16) | 24.85 (21.93 ,28.02) |  |
| Current smoker | 16.22 (14.37 ,18.26) | 25.98 (22.82 ,29.41) | 19.83 (16.35 ,23.85) |  |

Abbreviations: BMI: body mass index; SHBG: sex hormone-binding globulin;
For continuous variables: survey-weighted mean (95% CI) , P-value was by survey-weighted linear regression;
For categorical variables: survey-weighted percentage (95% CI) , P-value was by survey-weighted Chi-square test
